# Obesity and Wound Healing: Focus on Mesenchymal Stem Cells

**DOI:** 10.3390/life13030717

**Published:** 2023-03-06

**Authors:** Antonio Alma, Guya Diletta Marconi, Elena Rossi, Cristina Magnoni, Alessia Paganelli

**Affiliations:** 1 Dipartimento Chirurgico, Medico, Odontoiatrico e di Scienze Morfologiche con Interesse Trapiantologico, Oncologico e di Medicina Rigenerativa, University of Modena and Reggio Emilia, 41124 Modena, Italy; 2 Department of Innovative Technologies in Medicine & Dentistry, University “G. d’Annunzio” Chieti-Pescara, 66100 Chieti, Italy; 3 PhD Course in Clinical and Experimental Medicine, University of Modena and Reggio Emilia, 41124 Modena, Italy

**Keywords:** obesity, MSC, wound healing, ulcer, stem cell, adipose tissue, leptin, cytokine, diabetes, skin

## Abstract

Chronic wounds represent nowadays a major challenge for both clinicians and researchers in the regenerative setting. Obesity represents one of the major comorbidities in patients affected by chronic ulcers and therefore diverse studies aimed at assessing possible links between these two morbid conditions are currently ongoing. In particular, adipose tissue has recently been described as having metabolic and endocrine functions rather than serving as a mere fat storage deposit. In this setting, adipose-derived stem cells, a peculiar subset of mesenchymal stromal/stem cells (MSCs) located in adipose tissue, have been demonstrated to possess regenerative and immunological functions with a key role in regulating both adipocyte function and skin regeneration. The aim of the present review is to give an overview of the most recent findings on wound healing, with a special focus on adipose tissue biology and obesity.

## 1. Introduction

At present, obesity affects 1.7 billion people worldwide [1]. Notably, obesity often leads to a wide range of possible comorbidities, including cardiovascular disorders and diabetes (type 2 diabetes mellitus, T2DM, in particular) [2]. Most of these obesity-related diseases carry an intrinsic risk of developing chronic ulcers and/or causing significant impairment in physiological wound healing. For example, venous insufficiency is the commonest cause of chronic ulcers and is often associated to—and worsened by—obesity. Venous stasis may also determine delayed wound healing because of altered capillary flow due to impaired hydrostatic pressure [3,4,5]. Another frequent cause of chronic ulcers is peripheral artery disease (PAD), which is notably caused by atherosclerosis and therefore is, in turn, associated with obesity and/or T2DM. PAD determines a reduction in oxygen flow and nutrient supply, which are essential for tissue repair, and sometimes leads to the development of arterial ulcers through tissue ischemia. Neuropathy is another relatively common cause of cutaneous ulcers and represent a key driver for the development of chronic wounds in diabetic patients [6].

Not only do obesity and chronic ulcers represent significant health-related issues from a clinical and economic point of view, but they also lead to social and psychological consequences related to body image [7,8].

Obesity is by definition associated with an excess of adipose tissue. Adipose tissue is present in the human body in the form of brown and white adipose tissue (BAT and WAT), which are mainly responsible for thermogenesis and fat storage, respectively. However, brown adipocytes have been shown to possibly appear in WAT in response to specific thermogenic stimuli, therefore suggesting a more dynamic division between BAT and WAT and undermining previously consolidated notions of a static classification of adipose tissue subtypes [9]. Moreover, current research is pointing to adipose tissue as a more complex system, involved in several other physiological and/or pathological processes, including hormone metabolism, inflammation and wound healing [10,11].

Adipocyte dysfunction seems to play a role in obesity and its comorbidities, including chronic ulcers. For example, reduced adiponectin production by adipocytes has been described in obese patients and current evidence suggests a possible correlation with impaired wound healing [12]. At the same time, adipocytes represent a possible source of leukotriene B4, which prevents macrophage M2 polarization, therefore affecting the remodeling phase of wound healing [13]. Adipocyte lipolysis is also essential in wound repair [14], but it is not clear whether significant impairment is present in obese patients. Furthermore, inflammatory mediators and insulin-related signaling proteins, such as leptin and resistin, have been postulated to contribute to delayed wound healing in the setting of obesity [15].

Beyond adipocytes, other cell types are also present in the fibrous septa of the subcutaneous fat (e.g., endothelial cells, fibroblasts, inflammatory cells, etc.). Among these, ADSCs (adipose-derived mesenchymal stromal/stem cells) are of central importance for their role in wound healing. ADSCs are, in fact, adipose-tissue specific mesenchymal stromal/stem cells (MSCs) and are currently widely studied for their regenerative properties [11,16]. ADSCs have recently been demonstrated to be able to replace the dermal compartment and to promote wound re-epithelization [16,17,18,19]. ADSCs also seem to play a key role in the orchestration of the various phases of wound healing [14]. ADSC function is regulated by the tissue microenvironment: obesity, hypoxia and inflammation affect ADSC cellular plasticity and alter their immunophenotypic profile and regenerative functions [20]. In addition, obesity enhances their migratory potential and leads to their accumulation in visceral adipose tissue (VAT) [21].

**Figure 1:**
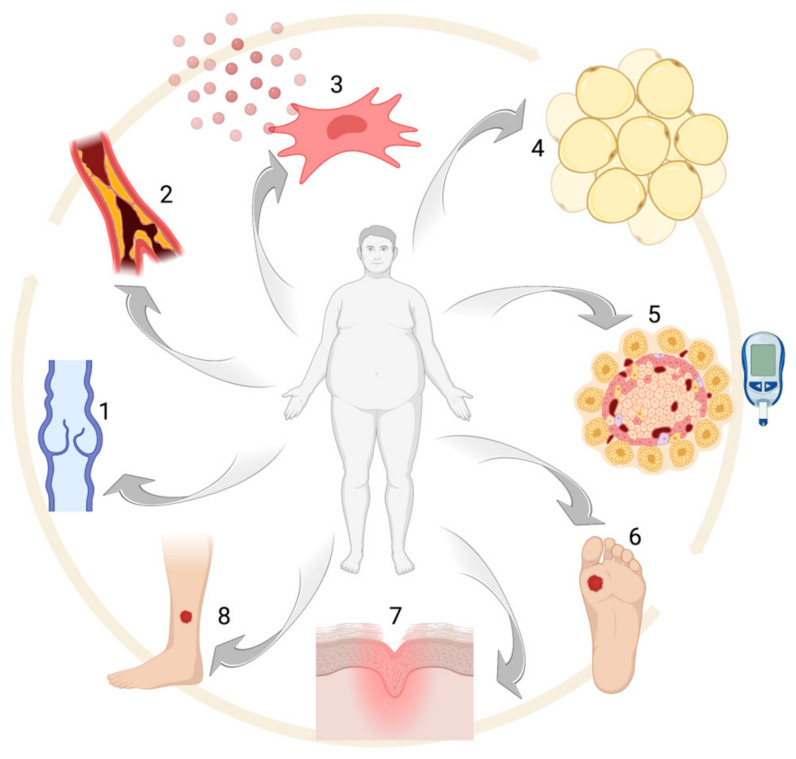
Obesity-associated alterations: (1) venous insufficiency, (2) atherosclerosis, (3) pro-inflammatory MSC phenotype, (4) adipocyte hypertrophy, (5) glucose intolerance and/or diabetes, (6–8) chronic ulcers. Created with BioRender.com.

The aim of the present study is to provide a complete overview of the most recent findings in the setting of wound healing and obesity, with a special focus on adipose tissue biology (see Figure 1).

## 2. Materials and Methods

A search was conducted in the PubMed/MedLine databases from their inception to the present. The following search terms were used: obesity, wound healing, MSCs, chronic ulcers, ADSCs. All the major journals were indexed. Articles without the full text electronically available and/or English translation were also excluded. After removal of papers not focusing on the link between MSCs, obesity and wound healing, we considered the articles referenced in the present review.

## 3. Obesity: Epidemiology and Comorbidities

Obesity is defined by the presence of a body mass index (BMI) ≥ 30 kg/m^2^, with normal range varying from 18.5 to 24.9 kg/m^2^. A BMI between 25.0 to 29.9 kg/m^2^ is considered overweight [22]. Obesity and being overweight are almost invariably caused by excessive caloric intake compared to the necessary amount, which in turns determines fat storage increase and adipocyte hypertrophy [23]. An unhealthy lifestyle is often the leading cause of obesity, but also genetic and epigenetic factors seem to play a very important role [24]. About 30% of the adult population in the world is overweight or obese, with western countries showing the highest prevalence. The Organization for Economic Cooperation and Development (OECD) estimated a prevalence for obesity ranging from 3.7% in Japan up to 38.2% in the US [25]. In the last two decades, the number of obese patients has tripled in Europe, where obesity is estimated to account for 7% of total healthcare costs [26]. However, the prevalence of obesity is nowadays constantly rising, even in developing countries [27,28], and rising childhood obesity rates portend worsening statistics [29].

According to the UK National Audit Office, obesity-related disorders cause significant loss in terms of both working days and deaths, with subsequent direct and indirect costs being estimated at approximately £480,000,000 and £2,150,000,000 per year, respectively [30]. In the U.S, the economic burden for obesity and its comorbidities was estimated to be around $147 billion in 2008 and $126 billion in 2016 [31,32].

Beyond these numbers, a large number of people is currently at risk of becoming obese, including children with familial history of obesity and/or metabolic syndrome, former smokers, lower social classes and older people [33,34,35,36]. As just mentioned, obesity is usually framed in the broader context of metabolic syndrome (or syndrome X), where it is associated with hypertriglyceridemia, atherosclerosis, reduced HDL, hypertension and impaired glucose tolerance [37]. In particular, metabolic syndrome is defined by the presence of three or more of the following criteria: (1) abdominal obesity (waist circumference ≥ 102 cm for men and ≥ 88 cm for women); (2) triglycerides ≥ 150 mg/dL; (3) high-density lipoprotein (HDL) cholesterol < 40 mg/dL for men and < 50 mg/dL for women; (4) systolic blood pressure ≥ 130 mmHg and/or diastolic blood pressure ≥ 85 mmHg and (5) fasting serum glucose ≥ 100 mg/dL [38].

One of the main issues in obese subjects is T2D, which is notably associated with insulin resistance [39,40,41]. Obese subjects also have an increased cardiovascular risk which, like other obesity-related comorbidities, can be explained by lipid accumulation in internal organs. Atherosclerosis, due to lipid accumulation in arterial walls, has got a pivotal role in coronary and cerebrovascular disease [42]. However, obesity also leads to increased platelet activation, which is responsible for thrombosis and subsequent further inflammation, increasing the likelihood of developing ischemic complications [40]. Triglyceride accumulation in the liver causes non-alcoholic fatty liver disease [43]. Moreover, obesity is a well-established risk factor for cholelithiasis due to cholesterol gallstones [44].

Obesity is also associated with a large variety of other possible comorbid conditions, including endocrine, oncological neurological, dermatological, respiratory and psychological disorders. Alterations in the hypothalamic–pituitary–gonadal (HPG) axis are often present in obese subjects [44]. In particular, polycystic ovary syndrome (PCOS) is strictly connected with metabolic syndrome, and weight loss is often part of the therapeutic regimen [45]. Obesity also carries a higher risk of developing several types of malignancies, such as colorectal, gastric, liver and gallbladder, endometrial and esophageal cancer [46].

As for the neurological complications, obese patients are more likely to develop small fiber sensory neuropathy (SFSN) [47] and recent studies suggest a higher risk of developing a form of cortical atrophy similar to Alzheimer’s disease (AD) [48].

Ulcers, lymphedema, intertrigo, hidradenitis suppurativa, striae distensae, skin tags, acanthosis nigricans, psoriasis, acne, hirsutism and androgenetic alopecia are the main skin condition connected to obesity and metabolic syndrome [49].

Biomechanical stress caused by a high body mass is responsible for numerous comorbidities of the musculoskeletal, respiratory, gastrointestinal and skin systems [44].

From a respiratory point of view, obesity finally increases the risk of obstructive sleep apnea syndrome (OSAS), chronic obstructive pulmonary disease (COPD) and asthma [50]. Even if not universally accepted, obesity is also associated with psychiatric/psychological conditions, including anxiety and depression [51].

Most of the aforementioned comorbidities are associated with the low-grade inflammation which nearly invariably comes with obesity. In fact, adipocytes can produce proinflammatory cytokines, such as IL-6, therefore maintaining the pro-inflammatory state typical of obese subjects, as also confirmed by elevated levels of c-reactive protein (CRP) in these patients [52,53]. This chronic inflammatory state is associated with hemodynamic and cardiac changes due to excessive body weight and contributes to the increased likelihood of having heart failure for obese subjects [54].

## 4. Wound Healing

**Figure 2:**
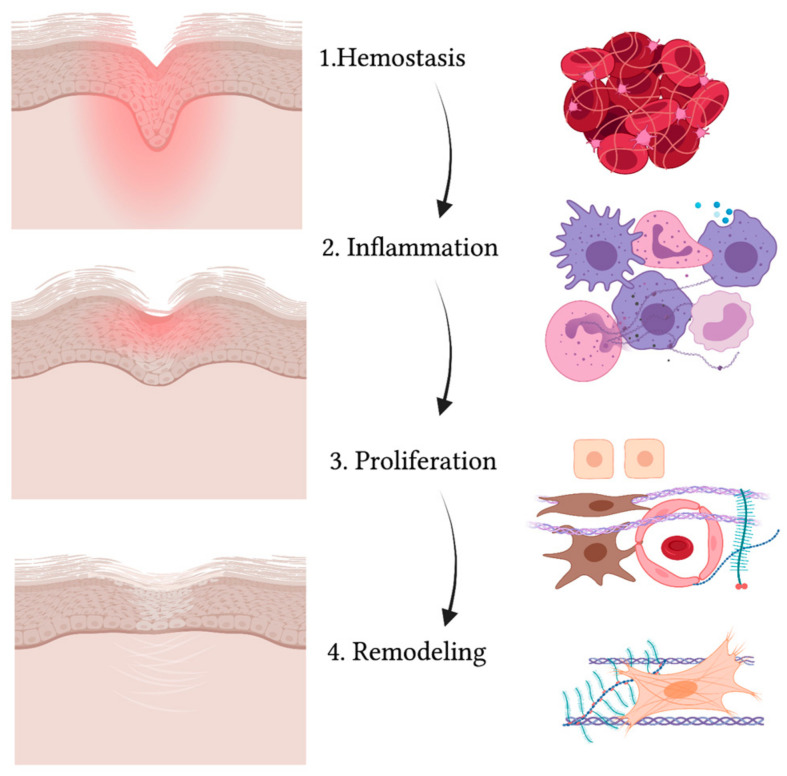
Schematic representation of the wound healing process. While the wound heals and the injured area (in red) reduces, gradually leading to a scar, the four phases take place (from upper to lower panel): hemostasis, inflammation, proliferation and remodeling. The main cell types are indicated on the right and include: red blood cells and platelets (1), leucocytes and professional phagocytes (2), keratinocytes (3), endothelial cells (3), MSCs (3) and fibroblasts (3,4). Created with BioRender.com.

A wound is defined as a disruption in the normal continuity of the skin. When the skin is injured, a series of events takes place in order to close and heal the area where the barrier is compromised [55]. Wound healing is an evolutionary-conserved process comprised of four sequential yet overlapping phases: hemostasis, inflammation, proliferation and remodeling (see Figure 2) [56]. These phases are strictly regulated through specific molecules that are expressed at different levels at each time interval [57].

The first process that takes place in the unwinding of wound healing consists of coagulation and hemostasis, aimed at stopping the bleeding while creating a temporary matrix for cells to infiltrate the site of injury [58]. In fact, not only does vascular smooth muscle contraction reduce the diameter of injured vessels as a mechanism of reflex, but also activation of the coagulation cascade allows platelets to form a clot with fibronectin, fibrin, vitronectin and thrombospondin [59]. Platelets also release several growth factors and cytokines when degranulating [60]. Growth factors and cytokines such as PDGF (platelet-derived growth factor), TGFβ (transforming growth factor β) and EGF (epidermal growth factor) activate neutrophils, macrophages, endothelial cells, fibroblasts and keratinocytes [61]. Then comes the inflammatory phase, where neutrophils, monocytes and macrophages flood to the site of injury [62]. Neutrophils help in removing cell debris and microorganisms that may have slipped into the wound via phagocytosis [63]. Macrophages also enter the site not only as professional phagocytes but also as regulatory cells secreting TGFα and TGFβ, HB-EGF (heparin-binding epidermal growth factor), FGFs (fibroblast growth factor) and collagenases [64,65]. Partially overlapping with the inflammatory phase, the proliferation stage is characterized by the activation, expansion and migration of fibroblasts, keratinocytes and endothelial cells [66]. Proliferation also involves the production of collagen, proteoglycans, hyaluronic acid and other ECM structural proteins that, along with fibroblast recruitment, give rise to granulation tissue [67]. Moreover, endothelial cells aid in forming new vessels and thus epithelization takes place [68]. Cytokines and growth factors activate keratinocytes so that they can migrate from the edges of the wound over the dermal matrix in order to close up the wound [69]. The expression of specific keratins, such as K6 and K16, is observed in migrating keratinocytes [70]. Lastly, collagenases, matrix metalloproteinases (MMPs) and their tissue inhibitors (TIMPs) are secreted by fibroblasts in the remodeling phase [65]. At this stage, fibroblasts differentiate towards a myofibroblast phenotype [71], and type III collagen is replaced by type I [72]. The remodeling phase is essential in order to restore skin elasticity as much as possible [66]. However, skin can only reach up to 80% of its original strength in a normal wound healing process and unavoidably heals with scarring, which represents the main difference between adult tissue repair and embryonic tissue regeneration [73,74].

Wounds can be either acute or chronic [75]. Acute wounds, such as surgical wounds, burns and skin tears, generally progress through the main phases of tissue repair and tend to heal quite easily within 6 weeks [76]. On the contrary, a prolonged inflammatory phase often accompanies chronic wounds due to a reduced nutrient supply, local hypoxic conditions and excess of exudates [62]. The presence of chronic ulcers and wounds can be associated with malnutrition, vitamin C deficiency, dehydration, autoimmune disorders, diabetes, arterial obstruction and/or venous congestion [77]. The vast majority of chronic wounds can be classified into a few major categories: venous and arterial ulcers, diabetic ulcers, pressure ulcers and post-surgical ulcers [78,79]. All these cases are associated by the need for the specific treatment of underlying causes and proper wound care in order to prevent wounds from remaining unhealed [80]. Healing may also be impaired and become exaggerated in keloid and hypertrophic scar formation, where excessive type III collagen formation in the proliferative phase causes an overgrowth of scar tissue [81]. To date, a lot of studies have been focusing on wound healing biology [82], and cell-based strategies are currently under investigation as possible therapies for skin regeneration [83,84].

## 5. Chronic Ulcers

During the last two decades, scientific interest for the wound healing setting has been constantly rising. Possible explanations for this reside in an increasing incidence of chronic ulcers, a larger population of elderly people and intensive medical care for chronic disorders [85]. Both older people and those who live with chronic diseases are at increased risk of developing chronic wounds [86]. Reduced mobility, age, diabetes, malnutrition and obesity are some of the clinical factors that can determine a delay in the wound healing process [87]. Chronic wounds are very debilitating morbid conditions, not only for the clinical and social consequences for patients but also for the related healthcare costs: nearly 2% of the US population is nowadays affected by chronic ulcers, accounting for a total cost of 20–25 billion USD per year [88,89]. It is estimated that the cost of wound care may account for 2–3% of the total healthcare budget in European countries. Moreover, from 27 to 50% of acute hospital beds are likely to be occupied on any day by patients with a wound [90]. Moreover, the psychological burden of living with chronic ulcers often implies reduced self-esteem, altered body image and, as a consequence, a reduced quality of life [91,92]. As already stated, chronic wounds can be due to a wide variety of possible causes, the most common being immobilization (pressure ulcers), arterial or venous insufficiency (arterial or venous ulcers), neuropathy (diabetic ulcers), ulcerated skin and/or soft-tissue neoplasms, with sometimes more than one cause being implicated in skin barrier disruption [93].

## 6. Obesity and Wound Healing

Wound healing seems to be impaired in obese patients compared to subjects with a normal BMI (body mass index), despite the mechanism underlying such a difference not being totally understood [94]. A multidisciplinary team is thus often essential for optimal wound care management in obese patients [89]. Decreased vascularization can partially explain wound healing delay [95]. In fact, the increase in the size of the adipocytes typical of obesity is generally not accompanied by an adequate rise in the number of vessels [96]. This eventually leads to a fibrotic environment [97], characterized by reduced elastin and increased collagen V and VI levels [98].

Wound healing disorders seem to have a huge impact in obese patients, both form a clinical and a social point of view. Venous insufficiency, caused or worsened by an elevated intra-abdominal pressure due to fat accumulation in the abdominal area [99], can cause leakage of proteinaceous-like material in the interstitial space, which can eventually occlude smaller vessels [100]. The resulting reduction in oxygen tension not only affects the proliferative and remodeling phases but also increases the risk of wound infection through the impairment of leukocyte phagocytic properties [12,101]. Arterial ulcers are associated to PAD, with atherosclerosis [89] being a well-known comorbidity in obese patients affected by metabolic syndrome [102] and/or T2D. Pressure ulcers are demonstrated to be common among obese patients staying in nursing homes [103]. However, contrasting data regarding the effects of obesity on the risk of development of pressure ulcers have been published so far in other patient subpopulations [104,105].

Peripheral neuropathy is another factor that can cause, aggravate or delay wound healing, and often represent the leading cause of cutaneous ulcers in diabetic patients [106,107]. Microangiopathy often represents the principal cause of impaired wound healing in T2D patients, leading to reduced nerve vascularization, endothelial dysfunction and impaired microcirculation [108]. On the other hand, macroangiopathy causes the production of prothrombotic factors and prevents the formation of an efficient network of collateral vessels, thus contributing to the pathogenesis of chronic wounds [109]. Finally, Advanced Glycation End-products (AGEs) appear to play a role in delaying wound healing in diabetic patients, affecting both angiogenesis and [110] extracellular matrix (ECM) production and remodeling [111]. Recent studies have demonstrated insulin to promote cell migration, and insulin-based therapies have therefore been suggested to surmount the impact of insulin resistance on wound repairing [111,112]. In addition, leptin, an anti-obesity hormone, improves wound repairing by accelerating angiogenesis and promoting the proliferation, differentiation and migration of keratinocytes [113]. Leptin is physiologically produced by adipocytes and leads to a reduction in the caloric intake, regulating body weight. Nevertheless, a high-fat diet leads to a leptin-resistant condition over time, thus limiting the effects of this hormone [114]. In obese patients, high leptin plasma levels are associated with peripheral receptor resistance [115].

## 7. MSCs

MSCs are defined as a subset of multipotent cells present in tissues of mesenchymal origin responsible for their regeneration [116,117]. MSCs were initially identified as spindle-shaped cells in the bone marrow (bone marrow stromal stem cells, BMSCs) [118,119,120]. Afterwards, other MSC reservoirs have been identified, including skeletal muscle, dental tissue, tendons, dermis, subcutaneous fat, liver, lungs, placenta, synovium, umbilical-cord, amniotic-fluid and breast milk [116,121,122,123,124,125,126]. The Mesenchymal and Tissue Stem Cell Committee of the International Society for Cellular Therapy identified three minimal defining criteria for MSCs: adherence to plastic, multipotency (ability to differentiate into chondrocytes, osteoblasts and adipocytes in vitro) and specific surface markers, such as CD105, CD73 and CD90 [127]. The absence of hematopoietic markers (e.g., CD34, CD45, CD14, CD19) is also crucial for identifying MSCs [127]. More recently, STRO-1, CD106 and CD146 have also been identified as MSC-defining markers [128,129]. Various authors already identified MSCs and their secretomes as key therapeutic tools in regenerative medicine, with possible applications in different medical settings, including—among others—neurology, orthopedics, dentistry and dermatology [130,131,132,133]. However, MSCs are currently studied not only for their regenerative properties, but also for their immunomodulating action [134]. Several mechanisms and molecules have been hypothesized so far for MSC-mediated immunoregulatory activity, involving both cell-to-cell contact and the secretion of soluble mediators [135,136]. For this reason, MSCs are currently under investigation for their potential therapeutic role in a wide range of autoimmune and/or autoinflammatory diseases [137,138,139]. Interestingly, aging seems to affect MSC function and to reduce their immunosuppressive activity [140]. Finally, a growing body of evidence also points at a possible pathogenetic role for MSCs in cancer, due to their anti-apoptotic, pro-angiogenic and immunosuppressive properties [141].

## 8. MSCs and Wound Healing

As already discussed, effective therapies for chronic wounds are urgently needed, not only to improve patient quality of life but to also reduce the burden of healthcare-related costs [142]. Despite advances in the context of wound care and the increasing employment of tissue-engineered skin substitutes in this setting, the results achieved with conventional techniques are still not completely satisfactory, and cell-based strategies are currently under investigation [143]. Adipose tissue and wound healing are closely related and a key role in this interplay is certainly played by ADSCs, fat-tissue resident MSCs. ADSCs are characterized by multipotency, plastic adherence and by the presence of specific surface molecules (CD73, CD90, CD105, CD106, CD146, STRO-1) in the absence of other lineage-specific markers [127,129,144]. ADSCs are particularly interesting for their relative abundance and the ease of the isolation procedure compared to other MSC subtypes [19,145,146]. Regenerative medicine and immune-mediated disorders certainly represent the principal applications for ADSC-based strategies [16,147,148,149,150,151,152]. As well as for other MSC sources, ADSC therapeutic functions are mostly mediated by the production of extracellular vesicles and the secretion of specific soluble mediators [153,154]. Several studies already demonstrated ADSC efficacy in the context of wound healing [155,156], therefore giving rise to a dedicated field of research.

Our group previously described the ability of ADSCs to secrete the ECM components and to produce an ADSC-based scaffolding material for the treatment of skin wounds [19,157]. Moreover, we demonstrated ADSC’s ability to promote re-epithelization in organotypic models through a direct action on basal stem keratinocytes [16]. Several authors also postulated that ADSCs themselves could differentiate into keratinocytes [158,159]. However, whether this is likely to happen in real-life remains to be determined, since basal and hair-follicle stem cells are considered to be physiologically more prone to contributing to re-epithelization [160] in the wound healing process. As well as for their immunomodulatory action, the regenerative and pro-epithelizing properties of MSCs seem to be mainly mediated by the secretion of soluble factors [161]. In order to prevent the potential oncogenic and immunogenic risks of direct cell transplantation [141], an increasing [67,130,162] trend in using ADSC-derived extracellular vesicles has been observed in the last few years, with exosomes being the most studied ones [163,164].

However, the use of ADSCs for the treatment of obesity-related disorders could not be limited to chronic wounds. In fact, ADSC-derived exosomes have been shown to inhibit both adipogenic differentiation and lipogenesis in ADSCs through activation of the Hedgehog (Hh) pathway [165,166]. Despite the promising results achieved with MSCs in the treatment of diabetes and its associated comorbidities, clinical data on the efficacy of autologous ADSCs are still lacking [167,168].

## 9. MSC Function and Obesity

**Figure 3:**
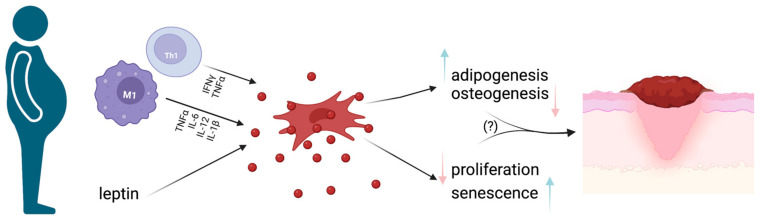
MSCs and obesity. Schematic representation of how the pro-inflammatory milieu found in obese subjects determines MSC impairment, with subsequent pro-inflammatory cytokine production, cell senescence and reduced proliferative ability. Such changes could be at least partially responsible for impaired wound healing and, subsequently, the occurrence of chronic wounds in obese subjects. Image created with BioRender.com.

MSCs’ regenerative properties strictly rely on their multipotency in terms of differentiation potential. MSC differentiation varies upon the environment where they are found and the specific stimuli they receive [122]. In particular, adipose differentiation is of central interest for a deeper understanding of obesity and its pathogenetic mechanisms. Despite MSC biology currently being widely studied, the intermediate stages between MSCs and terminally differentiated adipocytes are not well known. The chronic inflammatory milieu that characterizes obesity possibly affects MSC differentiation potential and, at a more local level, determines an increase in infiltrating macrophages (especially the M1 subtype) together with a significant decrease in Treg lymphocytes in WAT (See Figure 3) [11].

ADSC functions seem to be particularly affected by obesity, which has been demonstrated to lead to cell senescence [169]. As highlighted by a very recent review, obese subjects have increased ADSC content, but are characterized by lower proliferative ability, cell senescence and increased production of inflammatory cytokines [170]. A recent publication suggests that the presence of senescent ADSC in visceral WAT secreting pro-inflammatory and pro-aging factors could drive the typical increase in size and number of adipocytes observed in obese subjects [171]. Very recent studies also suggest a possible role for endocrine-disrupting chemicals (EDCs), such as bisphenol A (BPA), in inducing adipogenesis in ADSCs, therefore possibly contributing to the pathogenesis of obesity and its associated comorbidities [172,173]. An interesting study from Alessio [169] et al. clarified the molecular mechanisms implicated in MSC senescence, demonstrating the RB and p53 pathways to be activated in obese mice but not in controls. Moreover, the authors observed reduced proliferation rates in bone marrow and visceral fat MSCs but not in subcutaneous fat ADSCs, therefore suggesting substantial functional differences among the various WAT compartments of the body [169]. Other authors demonstrated obesity to be associated with BMSCs’ increased migratory activities. A Brazilian group demonstrated that [174] inflammatory cytokines—TNF alpha in particular—are able to induce the expression of CXCR4 on MSCs, which gives a plausible explanation of their enhanced migration in animal models of obesity. Moreover, leptin and interleukin-10 (IL-10) were found to be increased in visceral but not in subcutaneous WAT from obese mice, confirming site-dependent variations in the MSC microenvironment [21]. A high-fat diet is also demonstrated to promote adipogenesis and inhibit osteogenesis through leptin receptor signaling in bone marrow MSCs [175]. Consistent with these findings, an exercise-induced switch in MSC differentiation from adipogenesis to osteogenesis has been proposed as a potential biomarker for monitoring the effects of a healthier lifestyle on diabetes [176]. The fat content in umbilical cord-derived MSCs has been demonstrated to be an independent predictor of the offspring metabolic phenotype in terms of adiposity and fasting glucose levels [177]; such results confirm MSC alterations to be possibly involved in the development of obesity-related disorders.

MSC-based therapeutic protocols are widely studied in a large variety of diseases and have recently been explored as possible strategies for treating obesity-related disorders in pre-clinical models. For example, intraperitoneal injection of ADSCs was demonstrated to be effective in improving body weight and composition, glycemic control and lipid metabolism in obese mice [178]. The MSC therapeutic potential in this setting was also confirmed by another recent study using a murine model of obesity and T2D [179]. However, due to the significant functional impairment observed in obese subjects, the clinical efficacy of autologous MSCs in human diseases remains to be determined in this setting.

## 10. Conclusions

Obesity is currently a severe public health issue which raises the likelihood of suffering from concomitant morbid conditions including diabetes, cardiovascular diseases and, among others, chronic ulcers. Taken together, obesity-related disorders and chronic wounds represent major issues in human health, with significant burdens both in terms of healthcare costs and patient quality of life. The typical increase in terms of the visceral adipose tissue observed in obese subjects partially explains most of such possible comorbidities, due to its peculiar endocrine and immunological functions. In fact, WAT is nowadays recognized as being implicated in many different physiological processes beyond fat storage, including wound healing. However, adipose tissue also represents a strategic source for MSCs, which, in turn, play a pivotal role in both inflammation and tissue repair. Nevertheless, further studies are needed on the clinical efficacy of autologous ADSC-based strategies for the treatment of chronic wounds in obese subjects, due to the potentially detrimental effects of the subcutaneous pro-inflammatory milieu on MSC function.

## Data Availability

Data available from the authors upon reasonable request.
